# Diagnostic performance of deep learning–based reconstruction algorithm in 3D MR neurography

**DOI:** 10.1007/s00256-023-04362-z

**Published:** 2023-05-16

**Authors:** Falko Ensle, Malwina Kaniewska, Anja Tiessen, Maelene Lohezic, Jonas M. Getzmann, Roman Guggenberger

**Affiliations:** 1 Institute of Diagnostic and Interventional Radiology, University Hospital Zurich (USZ), University of Zurich, Raemistrasse 100, CH-8091 Zurich, Switzerland; 2 University of Zurich (UZH), Raemistrasse 100, CH-8091 Zurich, Switzerland; 3 GE HealthCare, Zurich, Switzerland

**Keywords:** 3D MR neurography, Deep learning-based reconstruction, Plexus

## Abstract

**Objective:**

The study aims to evaluate the diagnostic performance of deep learning–based reconstruction method (DLRecon) in 3D MR neurography for assessment of the brachial and lumbosacral plexus.

**Materials and methods:**

Thirty-five exams (18 brachial and 17 lumbosacral plexus) of 34 patients undergoing routine clinical MR neurography at 1.5 T were retrospectively included (mean age: 49 ± 12 years, 15 female). Coronal 3D T2-weighted short tau inversion recovery fast spin echo with variable flip angle sequences covering plexial nerves on both sides were obtained as part of the standard protocol. In addition to standard-of-care (SOC) reconstruction, *k*-space was reconstructed with a 3D DLRecon algorithm.

Two blinded readers evaluated images for image quality and diagnostic confidence in assessing nerves, muscles, and pathology using a 4-point scale. Additionally, signal-to-noise ratio (SNR) and contrast-to-noise ratios (CNR) between nerve, muscle, and fat were measured.

For comparison of visual scoring result non-parametric paired sample Wilcoxon signed-rank testing and for quantitative analysis paired sample Student’s *t*-testing was performed.

**Results:**

DLRecon scored significantly higher than SOC in all categories of image quality (*p* < 0.05) and diagnostic confidence (*p* < 0.05), including conspicuity of nerve branches and pathology. With regard to artifacts there was no significant difference between the reconstruction methods.

Quantitatively, DLRecon achieved significantly higher CNR and SNR than SOC (*p* < 0.05).

**Conclusion:**

DLRecon enhanced overall image quality, leading to improved conspicuity of nerve branches and pathology, and allowing for increased diagnostic confidence in evaluation of the brachial and lumbosacral plexus.

## Introduction

Three-dimensional (3D) sequences have become a mainstay in magnetic resonance (MR) neurography of the brachial and lumbosacral plexus [1]. This is owed to their large spatial coverage with ability to obtain multiplanar reformations and maximal intensity projections (MIP), allowing complete depiction of nerve fibers along their longitudinal axis [2]. Consequently, they are helpful to not only illustrate morphology to referring physicians and aid in preoperative planning but also to detect subtle pathologic findings [3]. To fully leverage these capabilities of 3D MR neurography, image quality is paramount because of the complex anatomy and small caliber of plexus branches.

However, 3D isotropic acquisition comes with a loss in signal-to-noise ratio (SNR) and spatial resolution compared to two-dimensional (2D) imaging, given equivalent scan times [4]. Previously this challenge has been addressed with advanced acceleration techniques such as parallel imaging and compressed sensing, in order to reduce scan time or increase spatial resolution [5, 6]. However, using high acceleration rates can in turn lead to significant deterioration of SNR as a result of undersampling and noise amplification [7], potentially masking relevant image details. An innovative and promising approach to counter this tradeoff is the emerging introduction of artificial intelligence (AI) in image reconstruction. Applying deep learning (DL) reconstruction to 3D MR neurography could improve image quality by compensating for the mentioned drawbacks in resolution and SNR. Inversely it may have the potential to decrease scan times while preserving adequate image quality.

Such a reconstruction algorithm, DLRecon (AIR^™^ Recon DL, GE HealthCare, Waukesha, WI, USA), was recently evaluated in 2D MR neurography of the extremities, where it showed improved conspicuity of key morphological features of the nerves [8]. Employing this AI method in 3D MR neurography may even further increase its effect on improving nerve depiction, since 3D data generally yields higher SNR than 2D.

Although DL image reconstruction of 2D MRI has recently been introduced on some vendor platforms, its clinical use in 3D acquisitions remains largely unexplored and requires comparison of diagnostic performance prior to routine implementation.

We hypothesized that DLRecon would enhance overall image quality, enabling increased diagnostic confidence in the evaluation of the brachial and lumbosacral plexus. The objective of our study was to evaluate the diagnostic performance of deep learning–based reconstruction algorithm in 3D MR neurography for the assessment of the brachial and lumbosacral plexus.

## Materials and methods

### Study design

This study was approved by the institutional review board (BASEC-Nr. 2021-02408). Written general consent was obtained from all participants prior to imaging.

Forty-four consecutive exams of the brachial and lumbosacral plexus of 41 patients were included in this retrospective study between January 2022 and September 2022. Nine exams could not be reconstructed with the DL algorithm due to incomplete datasets, resulting in *n* = 35 exams (18 brachial and 17 lumbosacral plexus) of 34 patients for final analysis (mean age: 49 ± 12 years, 15 female). One female patient had a scan of both the brachial and the lumbosacral plexus. Patients were referred with clinical diagnosis or suspicion of the following (*n*): polyneuropathy (8), post-traumatic neuropathy (6), chronic inflammatory demyelinating polyneuropathy (CIDP) (4), post-surgical neuropathy (4), neoplastic neuropathy (4), radiculopathy (3), unspecific neuropathy (3), and Parsonage-Turner syndrome (2). Demographics of study participants are shown in Table 1.

**Table 1:** Study participant demographics

Characteristic	Value
Total examinations	35
Brachial plexus	18
Mean age ± standard deviation	50 ± 11 years
Males/females	12/6
Lumbosacral plexus	17
Mean age ± standard deviation	47 ± 14 years
Males/females	8/9

All patients who presented to our institution for standard-of-care MR plexus neurography were considered for study inclusion. Exclusion criteria were insufficient image quality due to motion or metal hardware artifacts, inability to reconstruct images with the DL algorithm, and patient age <18 years.

### Image acquisition

All examinations were performed on a clinical 1.5-T MRI System (SIGNA^™^ Artist, GE HealthCare, Waukesha, WI, USA). Patients were scanned in supine position using a combined coil setup for the lumbosacral plexus of a 30-channel anterior array coil and a 40-channel posterior array. For the brachial plexus a 19-channel head/neck coil was added. Coronal 3D T2-weighted short tau inversion recovery fast spin echo with variable flip angles (“CUBE-STIR”) sequences were obtained covering both sides as part of the institution’s standard MR plexus neurography protocol, before administration of gadolinium intravenous contrast.

Pulse sequence parameters are listed in Table 2. No compressed sensing acquisition or parallel imaging was utilized.

**Table 2:** Parameters of 3D STIR-FSE CUBE sequence

Parameter	Brachial plexus	Lumbosacral plexus
Field of view (FOV) (cm)	32	38
Matrix (frequency× phase)	268 × 268	288 × 282
Slice thickness (mm, 0.8-mm gap)	1.6	1.6
Number of slices (median)	116	135
Inversion time (TI) (ms)	180	180
Repetition time (TR) (ms)	~2802	~2602
Echo time (TE) (ms)	65	65
Bandwidth (kHz)	42	50
Excitations (NEX)	1	1
Echo train length	96	104
Median scan time (min:s)	4:33	5:08

### Deep learning image reconstruction

Conventional reconstruction was used in routine clinical practice and is termed standard-of-care (SOC) in this study. In addition, the raw acquired data of the MRI exams was retrospectively reconstructed offline with a vendor-supplied prototype of a deep convolutional neural network, i.e., AIR^™^ Recon DL 3D (GE HealthCare, Waukesha, WI, USA) [9, 10]. DLRecon is a deep learning–based reconstruction pipeline tightly integratable in the scanner’s native reconstruction pipeline, which takes raw *k*-space data as its input and generates high-fidelity images as output [8, 11]. It is designed to perform denoising, reduce truncation artifacts, and improve edge sharpness. The tested prototype is an extension of the commercially available 2D version of AIR Recon DL, making it applicable to 3D data.

The prototype allows to customize the degree of noise reduction through a scalable noise reduction factor ranging from 0 to 100%. After reviewing a preliminary reconstruction sample with varying noise reduction factors, we chose the highest setting (100%) in order to leverage the algorithm’s full potential.

### Image analysis

#### Qualitative analysis

Two readers (a board-certified radiologist with 7 years and a radiology resident with 4 years of experience) independently evaluated all images at random on a picture archiving and communication system (PACS) (DeepUnity Diagn, Dedalus, Bonn, Germany) with diagnostic quality monitors. The sequences were extracted from the patients’ imaging files and anonymized. Both readers were blinded to the technique, clinical information, and the radiological report. Prior to independent grading, the readers jointly reviewed images not included in the study sample and discussed discrepancies until consensus was reached.

In each exam, the entire imaging volume was assessed in all three planes using a multiplanar reconstruction tool in PACS. Additionally, standardized coronal MIPs with a slab thickness of 10 mm were generated beforehand for evaluation.

A 4-point scale (0—poor, 1—moderate, 2—good, 3—perfect) was used to visually score the following parameters: (1) contour sharpness of plexus; (2) plexus signal homogeneity; (3) contrast of plexus to surrounding tissue; (4) fat suppression quality; (5) muscle signal homogeneity; (6) diagnostic confidence in muscle evaluation; (5) overall image quality; and (6) overall diagnostic confidence in nerve evaluation. Furthermore, the suprascapular and obturator nerves in the brachial and lumbosacral plexus, respectively, were separately evaluated for conspicuity and diagnostic confidence. We chose to assess those branch nerves as they are most reliably identified and commonly part of the clinical question in our routine practice, for example, in the setting of suspected Parsonage-Turner syndrome or obturator neuropathy [12, 13]. Muscle analysis was included, because 3D STIR-FSE sequences afford a large field of view to screen for denervation changes like edema or atrophy as important markers of neuropathy [14].

A subset of exams in which an abnormality was identified in the initial radiology report (*n* = 8) was assessed by the blinded readers for conspicuity of pathology (lesion conspicuity) and diagnostic confidence. This subset was also excluded from further quantitative analysis.

The presence of artifacts was also graded on a 4-point scale (0—none, 1—mild, 2—moderate, 3—severe), based on their detrimental effect on image evaluation.

#### Quantitative analysis

To quantitatively compare image quality of the reconstruction methods, signal-to-noise ratio (SNR) and contrast-to-noise ratio (CNR) of nerve, muscle and fat were calculated. The subset with pathology (*n* = 8) was excluded from quantitative analysis, to avoid potential distortion of the DLRecon effect. Manually drawn 5-mm^2^ regions of interest were placed by a radiographer with 11 years of experience in the following locations: In brachial plexus C6, C7, and C8 nerve within interscalene triangle, adjacent middle scalene muscle, retroclavicular plexus portion, fat in supraclavicular region, and extracorporeal background air and in lumbosacral plexus post-ganglionic L5 nerve bilaterally, femoral nerve in iliac fossa, adjacent iliac muscle, sciatic nerve at level of ischial spine, fat in gluteal region, and extracorporeal background air.

The mean of all 4 nerve measurements in the brachial and lumbosacral plexus, respectively, served as nerve signal for the SNR and CNR, which were calculated as:

$$SNR=\frac{SI}{SD(air)}$$

$$CNR=\frac{SI- SI}{SD(air)}$$

### Statistical analysis

Variables are reported as mean ± standard deviation (SD). For comparison of visual scoring result non-parametric paired sample Wilcoxon signed-rank testing, for quantitative analysis paired sample Student’s *t*-testing was performed. Analysis of inter-rater agreement within reconstruction methods was conducted with Cohen’s kappa (*κ*). According to Landis and Koch [15], degree of agreement was considered poor (*κ* < 0), slight (*κ* = 0.00–0.20), fair (*κ* = 0.21–0.40), moderate (*κ* = 0.41–0.60), substantial (*κ* = 0.61–0.80), or near-perfect (*κ* = 0.81–1.00). Statistical significance was set a priori to *p* < 0.05.

All statistical analyses were performed using proprietary software (SPSS software version 21.0, IBM, Armonk, NY).

## Results

### Qualitative analysis

Table 3 provides a full summary of the scoring results for image quality and diagnostic confidence.

**Table 3:** Image quality and diagnostic confidence scoring results between standard-of-care (SOC) and deep learning (DLRecon) reconstruction method

	Plexus brachialis (mean ± SD)		Plexus lumbosacralis (mean ± SD)		*p*-Value
Metric	SOC	DLRecon	SOC	DLRecon	
Image quality					
Contour sharpness of plexus	1.5 ± 0.5	2.4 ± 0.5	1.5 ± 0.6	2.7 ± 0.6	<0.001–0.003
Plexus signal homogeneity	1.4 ± 0.5	2.3 ± 0.6	1.6 ± 0.6	2.7 ± 0.8	
Contrast of plexus to surrounding tissue	1.6 ± 0.6	2.4 ± 0.7	1.6 ± 0.7	2.6 ± 0.8	
Fat suppression quality	1.7 ± 0.5	2.7 ± 0.5	2 ± 0.5	2.9 ± 0.2	
Muscle signal homogeneity	1.7 ± 0.5	2.7 ± 0.5	2 ± 0.5	2.9 ± 0.3	
Overall image quality	1.4 ± 0.5	2.3 ± 0.7	1.5 ± 0.6	2.5 ± 0.8	
Conspicuity N. suprascapularis/obturatorius	0.7 ± 0.8	1.7 ± 1.0	1.1 ± 1.2	1.8 ± 1.3	
Overall artifacts	1.3 ± 0.6	1.1 ± 0.8	1.3 ± 0.6	1.2 ± 0.6	0.102/0.157
Diagnostic confidence					
Muscle	2.2 ± 0.8	2.8 ± 0.4	2.5 ± 0.7	2.9 ± 0.2	<0.005
Plexus proper	1.4 ± 0.5	2.3 ± 0.7	1.6 ± 0.7	2.5 ± 0.8	<0.001
N. suprascapularis/obturatorius	0.7 ± 0.8	1.6 ± 1.1	1.1 ± 1.1	1.7 ± 1.4	<0.001/0.004

DLRecon = deep learning reconstruction, N. = nervus, SD = standard deviation, SOC = standard of care

DLRecon scored significantly higher than SOC in all image quality features (*p* < 0.003), except in the artifact category, where there was no significant difference between the reconstruction methods (*p* = 0.102 and 0.157 for brachial and lumbosacral plexus, respectively).

With regard to diagnostic confidence, DLRecon also received significantly higher scores than SOC in all categories.

Inter-rater agreement for image quality categories in DLRecon ranged from moderate to near-perfect in lumbosacral plexus (*κ* = 0.59–0.88) and moderate to substantial in brachial plexus (*κ* = 0.56–0.80); inter-rater agreement in SOC was moderate to substantial (*κ* = 0.54–0.78 and 0.59–0.78 for brachial and lumbosacral plexus, respectively).

Evaluation of diagnostic confidence in DLRecon demonstrated moderate to near-perfect inter-rater agreement for brachial plexus (*κ* = 0.6–0.82) and substantial for lumbosacral plexus (*κ* = 0.64–0.78). In SOC, inter-rater agreement was moderate to substantial for brachial plexus (*κ* = 0.57–0.7) and substantial for lumbosacral plexus (*κ* = 0.61–0.75). Table 4 contains a detailed overview of the individual inter-rater agreement values.

**Table 4:** Inter-rater agreement Cohen’s kappa values for standard-of-care (SOC) and deep learning (DLRecon) reconstruction method

	Plexus brachialis(*κ*)		Plexus lumbosacralis(*κ*)	
Metric	SOC	DLRecon	SOC	DLRecon
Image quality				
Contour sharpness of plexus	0.60	0.57	0.59	0.69
Plexus signal homogeneity	0.56	0.69	0.61	0.59
Contrast of plexus to surrounding tissue	0.72	0.75	0.67	0.88
Fat suppression quality	0.61	0.64	0.64	0.64
Muscle signal homogeneity	0.72	0.73	0.62	0.60
Overall image quality	0.78	0.72	0.72	0.69
Conspicuity N. suprascapularis/obturatorius	0.57	0.80	0.73	0.73
Overall artifacts	0.54	0.56	0.78	0.74
Diagnostic confidence				
Muscle	0.7	0.82	0.61	0.64
Plexus proper	0.61	0.62	0.75	0.78
N. suprascapularis/obturatorius	0.57	0.6	0.72	0.71

DLRecon = deep learning reconstruction, N. = nervus, SOC = standard of care, *κ* = Cohen’s kappa

Based on imaging reports, a total of 8 exams (6 brachial and 2 lumbosacral plexus) showed nerve pathologies: CIDP (*n* = 3), post-traumatic neurotmesis (*n* = 1), post-radiogenic plexopathy (*n* = 1), C7 radiculopathy (*n* = 1), unspecific plexopathy (*n* = 1), and Pancoast-tumor infiltration (*n* = 1). MRI findings correlated with sonographic (*n* = 3), electrodiagnostic (*n* = 2), and clinical exam (*n* =3) findings in all cases.

When evaluating these exams with identified nerve pathologies, DLRecon significantly improved lesion conspicuity and diagnostic confidence compared to SOC (*p* = 0.046 and 0.014, respectively). The mean ± standard deviation of review scores from SOC and DLRecon were 2.1 ± 0.6 and 2.7 ± 0.5 for lesion conspicuity and 1.9 ± 0.6 and 2.6 ± 0.5 for diagnostic confidence. Inter-rater agreement for lesion conspicuity was substantial in both DLRecon and SOC (*κ* = 0.65 and 0.67, respectively), whereas assessment of DLRecon improved inter-rater agreement for diagnostic confidence (*κ* = 0.75) compared to SOC (*κ* = 0.58).

**Fig. 1:**
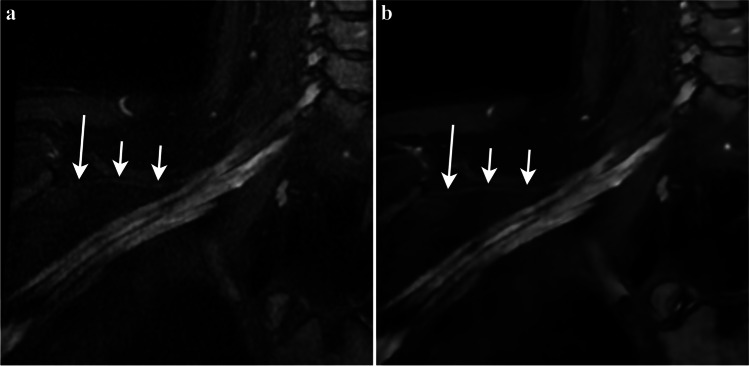
Thirty-four-year-old man with suspected CIDP. Three-dimensional STIR-FSE multiplanar reformation (MPR) reconstructed with the SOC method (**a**) shows a faintly visible suprascapular nerve (arrows). The distal aspect of the nerve (long arrow) is difficult to identify, largely due to low SNR. On the 3D STIR-FSE MPR reconstructed with the DLRecon method (**b**) the suprascapular nerve, normal in appearance, is clearly delineated, allowing for confident evaluation to the periphery. Note also the diffuse enlargement and signal hyperintensity of the supraclavicular and infraclavicular plexus, consistent with CIDP. CIDP = chronic inflammatory demyelinating polyneuropathy, DLRecon = deep learning reconstruction, MPR = multiplanar reformation, SNR = signal-to-noise ratio, SOC = standard of care, STIR-FSE = short tau inversion recovery fast spin echo

**Fig. 2:**
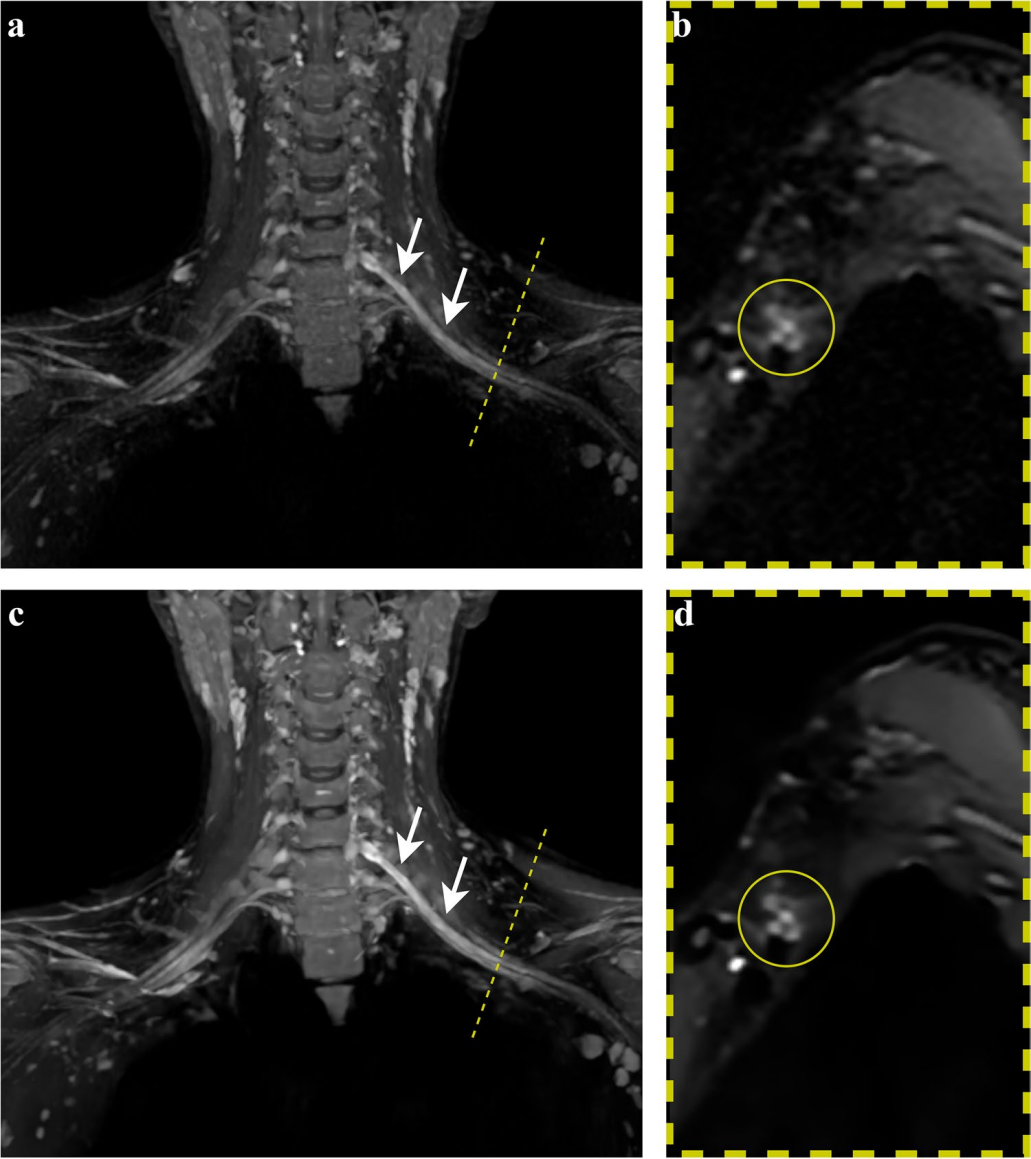
Thirty-nine-year-old woman with non-specific left C7 radiculopathy. Three-dimensional STIR-FSE coronal MIP (left pictures) demonstrates thickening and signal hyperintensity of the left extraforaminal C7-nerve root propagating into the middle trunk (arrows) and the corresponding divisions, as seen in sagittal reformation (right pictures) at retroclavicular level (dashed line). Images processed with DLRecon (**c**, **d**) exhibit superior conspicuity of pathology on the coronal MIP and allow clearer separation of the individual plexus structures on the sagittal reformat (circle) compared to the SOC reconstruction method (**a**, **b**). DLRecon = deep learning reconstruction, MIP = maximum intensity projection, SOC = standard of care, STIR-FSE = short tau inversion recovery fast spin echo

**Fig. 3:**
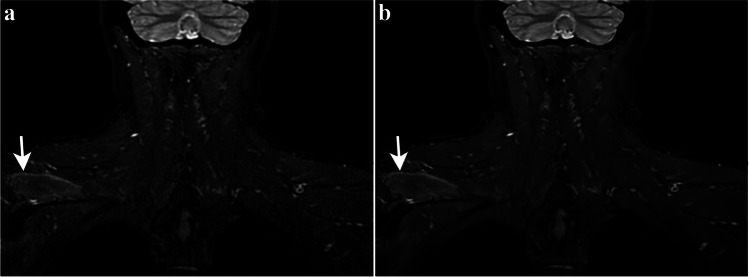
Fifty-year-old man with right arm paralysis following a skiing accident. Coronal 3D STIR-FSE image shows denervation edema pattern of the right supraspinatus muscle (arrow) with overall more homogeneous signal and sharper delineation of the muscles in the DL reconstruction (**b**) compared to the SOC reconstruction (**a**). DL = deep learning, SOC = standard of care, STIR-FSE = short tau inversion recovery fast spin echo

**Fig. 4:**
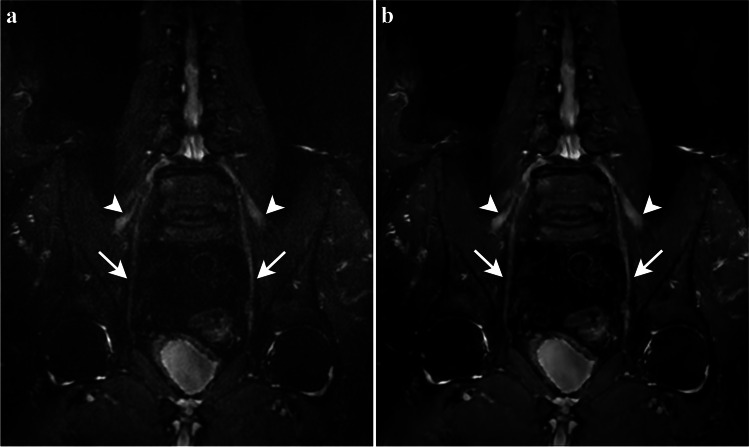
Seventy-two-year-old man with suspected CIDP. Coronal 3D STIR-FSE image displays irregular enlargement and signal hyperintensity of the femoral (arrowheads) and obturator (arrows) nerve bilaterally, consistent with CIDP. SOC reconstruction (**a**) demonstrates noise and partly blurred contours, notably of the right obturator nerve. Reconstruction with DLRecon (**b**) results in marked noise reduction and improved sharpness, making the irregular fusiform enlargement of the obturator nerve more conspicuous. CIDP = chronic inflammatory demyelinating polyneuropathy, DLRecon = deep learning reconstruction, SOC = standard of care, STIR-FSE = short tau inversion recovery fast spin echo

**Fig. 5:**
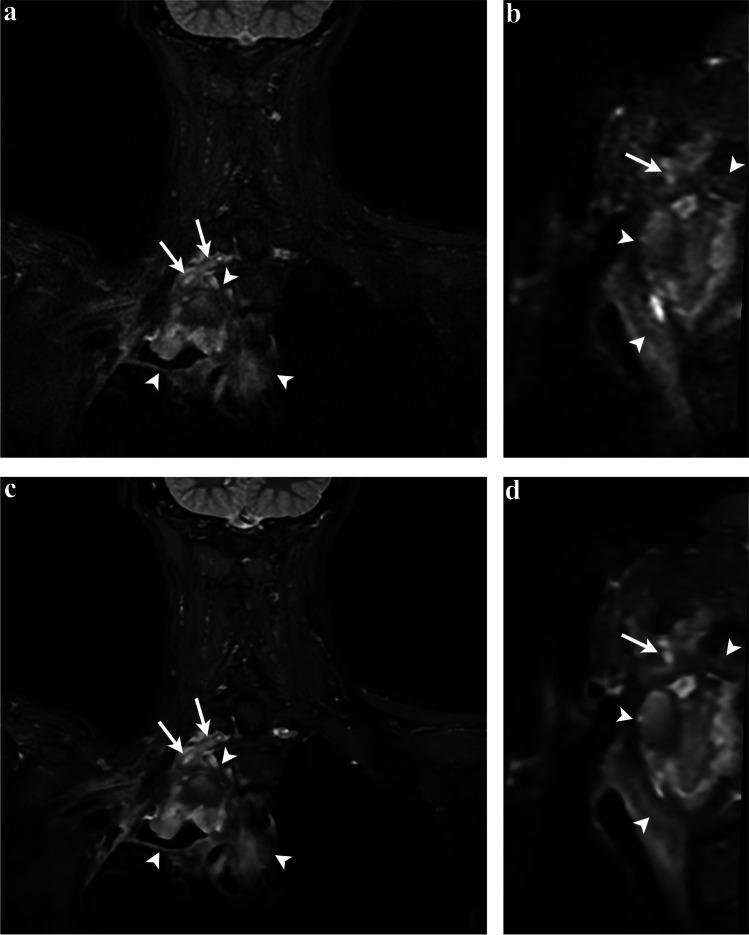
Fifty-six-year-old woman with Pancoast tumor post-radiotherapy. Three-dimensional STIR-FSE images show a cavitary Pancoast tumor in the right lung apex (arrowheads) with encasement of the thickened and hyperintense T1 nerve (arrows). DLRecon demonstrates sharper delineation of the tumor margins and the nerve on the coronal image (**c**) and sagittal MPR (**d**), compared to SOC reconstruction (**a** and **b**, respectively). DLRecon = deep learning reconstruction, SOC = standard of care, STIR-FSE = short tau inversion recovery fast spin echo

Exemplary images of 3D plexus depiction with DLRecon and SOC are demonstrated in Figs. 1, 2, 3, 4, and 5.

### Quantitative analysis

**Fig. 6:**
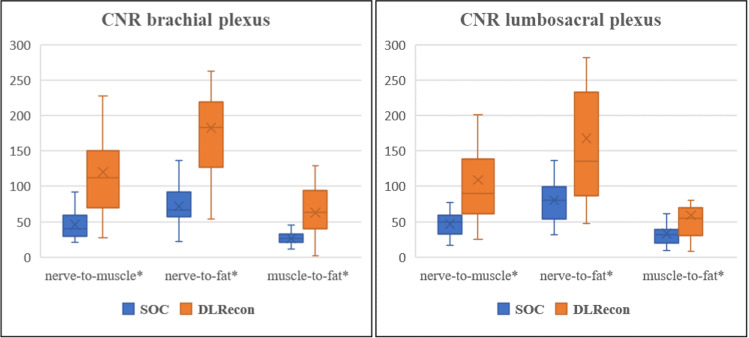
Box and whisker plot comparing contrast-to-noise ratio (CNR) between the standard-of-care (SOC) and deep learning (DLRecon) reconstruction method (* mark: *p* < 0.05). CNR = contrast-to-noise ratio, DLRecon = deep learning reconstruction, SOC = standard of care

DLRecon achieved significantly higher CNR and SNR than SOC in both the brachial and lumbosacral plexus, as illustrated in Fig. 6 for CNR.

#### Brachial plexus

Mean ± standard deviation of CNR from DLRecon and SOC were 120.2 ± 73.8 and 45.7 ± 19.6 for nerve-to-muscle, 182.8 ± 91.5 and 72.1 ± 26.4 for nerve-to-fat, and 62.6 ± 31.9 and 26.3 ± 11.7 for muscle-to-fat. SNR were 234.2 ± 115.8 and 94.2 ± 33.5 for nerve, 114.0 ± 50.1 and 48.5 ± 17.2 for muscle, and 51.4 ± 29.7 and 22.1 ± 9.2 for fat.

The associated *p* values for CNR and SNR were all less than 0.001.

#### Lumbosacral plexus

The mean ± standard deviation of CNR from DLRecon and SOC were 108.9 ± 75.4 and 47.0 ± 17.7 for nerve-to-muscle (*p =* 0.002), 167.8 ± 113.9 and 80.1 ± 30.5 for nerve-to-fat (*p =* 0.003), and 58.9 ± 45.1 and 33.1 ± 18.0 for muscle-to-fat (*p =* 0.002).

SNR were 211.5 ± 129.9 and 106.4 ± 40.2 for nerve (*p =* 0.003), 102.5 ± 60.5 and 59.4 ± 27.0 for muscle (*p =* 0.006), and 43.6 ± 18.9 and 26.3 ± 11.9 for fat (*p =* 0.001).

## Discussion

In this study, we applied DLRecon to 3D MR neurography of the brachial and lumbosacral plexus, comparing it to conventional SOC reconstruction method. DLRecon demonstrated a significant improvement in qualitative and quantitative assessment of the plexus. Along with superior image quality, DLRecon allowed for significantly improved conspicuity of nerve branches and pathology. Consequently, diagnostic confidence in evaluating nerves, muscles, and pathology was also significantly enhanced. The independent reader analyses of the reconstructed images were concordant with the objective quantitative measures, reflecting the expected properties of the reconstruction algorithm, i.e., denoising and image sharpening.

These results support the hypothesis that DL-based reconstruction may help mitigate the shortcomings of 3D STIR-FSE images in SNR and spatial resolution at reasonable scan times. Adequate image quality is essential for examining the plexus with high diagnostic accuracy, due to complex anatomy and small caliber of nerve branches. Despite technological advancements through development of acceleration, vascular suppression, and 3D techniques [16], MR neurography remains a challenging field of musculoskeletal imaging while playing an increasing role in the diagnosis and management of peripheral neuropathies [17, 18].

Our findings are in line with a recent study by Sneag et al., which demonstrated efficacy of the same DL algorithm, yet in 2D MR neurography of the extremities at 3 T [8]. In that study, DLRecon increased conspicuity of morphological features critical to evaluating a nerve injury, i.e., outer epineurium and fascicular architecture. Further, improved inter-rater agreement with DLRecon was reported. In our study on 3D DLRecon images at 1.5 T, inter-rater agreement varied between reconstruction methods, as a wider range of variables were assessed, e.g., including muscles, nerve pathology, and diagnostic confidence.

DLRecon also improved conspicuity of the suprascapular and obturator nerves. This can be mainly attributed to denoising of the algorithm, which led to lower noise in the surrounding fat area and causes an apparent reduction of fat signal. Visualizing these small plexus branches is a key quality feature of MR neurography, as their evaluation is often part of the clinical question in routine practice.

Furthermore, nerves were sharper delineated in DLRecon, which could improve detection or exclusion of focal abnormalities like subtle intrinsic constrictions in Parsonage-Turner syndrome. To that end, 2D sequences are still relied upon to provide high in-plane spatial resolution. However, confident evaluation of plexus branches is often impeded by their non-linear course, whereas isotropic 3D imaging can depict the nerves along their entire longitudinal extent without partial-volume averaging. Ultimately, AI-enhanced 3D sequences could even partly replace 2D sequences in MR neurography in the future, leading to substantial overall time savings.

No significant difference in artifacts was observed between DLRecon and SOC, even though the algorithm is specifically designed to remove truncation artifacts. However, we graded artifacts that mostly appeared in the extracorporal air or in the periphery of the anatomy regarding their effect on image interpretation. On average, artifacts were rated as mild in both the brachial and lumbosacral plexus as they had little impact on diagnostic yield.

In this study, we have demonstrated efficacy of 3D DLRecon at 1.5-T MR neurography. Before the advent of AI reconstruction techniques, some authors have advocated the use of 3 T in the past [19]. With widespread implementation of AI-based image reconstruction enhancing its image quality, state-of-the-art MR neurography should now also be routinely achievable at 1.5 T with adequate high quality.

While this study has demonstrated the benefits of the DLRecon algorithm, we acknowledge several limitations. The study contained a modest sample size of 35 exams in two varying anatomical regions. In particular, the subset with identified pathologies was small and contained a heterogenous set of abnormalities. Additionally, there was no correlation of MRI findings with an electrodiagnostic reference standard; nevertheless, high correlation has been described in previous studies for diagnosis of peripheral neuropathy [20–23].

Despite best efforts to perform a blinded reader study, the image features of DLRecon were distinctive, such that the reconstruction status was likely noticeable to study raters and may have inflicted some bias on the scoring. Anecdotally, readers perceived apparent noise removal and image sharpening in DLRecon compared to SOC images.

In summary, our findings suggest that DLRecon improves overall image quality including clinically relevant imaging features, enabling increased diagnostic confidence in the evaluation of the brachial and lumbosacral plexus. The DLRecon method could enhance diagnostic performance/yield and add clinical value to the assessment of suspected neuropathy. Further research is necessary to quantify the clinical benefit compared to SOC image reconstruction.

## Abbreviations

AI: Artificial intelligence
CIDP: Chronic inflammatory demyelinating polyneuropathy
CNR: Contrast-to-noise ratio
DL: Deep learning
FOV: Field of view
MIP: Maximal intensity projection
MRI: Magnetic resonance imaging
PACS: Picture archiving and communication system
SD: Standard deviation
SNR: Signal-to-noise ratio
SOC: Standard of care
STIR-FSE: Short tau inversion recovery fast spin echo
2D: Two-dimensional
3D: Three-dimensional
